# Assessing the Comparability of Degradation Profiles Between Biosimilar and Originator Anti-VEGF Monoclonal Antibodies Under Thermal Stress

**DOI:** 10.3390/ph18091267

**Published:** 2025-08-26

**Authors:** Ceren Pamukcu, Ahmet Emin Atik

**Affiliations:** 1 Biotechnology Group, Turgut Ilaclari A.S., 41400 Kocaeli, Türkiye; cerenpamukcu93@gmail.com; 2 Department of Natural Sciences, Faculty of Engineering and Natural Sciences, Acibadem Mehmet Ali Aydinlar University, 34752 Istanbul, Türkiye

**Keywords:** forced degradation, biosimilarity assessment, orthogonal analytical techniques, degradation profile, thermal stress

## Abstract

**Background/Objectives**: Forced degradation studies are critical for identifying potential degradation pathways of monoclonal antibodies (mAbs), particularly under thermal stress. Due to their structural complexity and sensitivity to elevated temperatures, mAbs are prone to fragmentation, aggregation, and post-translational modifications. This study aimed to evaluate and compare the degradation profiles of biosimilar anti-VEGF mAb and its originator counterparts sourced from both the United States (U.S.) and the European Union (EU) under thermal stress conditions. To our knowledge, this represents one of the few studies conducting a direct head-to-head comparability assessment across biosimilar and two geographically sourced originators, integrating orthogonal analytical approaches. **Methods**: Biosimilar candidate and originator products (U.S. and EU) were incubated at 37 °C and 50 °C for 3, 7, and 14 days. Fragmentation profiles were assessed using validated non-reduced and reduced capillary electrophoresis–sodium dodecyl sulfate (CE-SDS) methods. Additionally, size-exclusion ultra-performance liquid chromatography (SE-UPLC) and liquid chromatography–tandem mass spectrometry (LC-MS/MS) assays were performed on samples stressed for 14 days to provide deeper insights into degradation pathways. **Results**: Non-reduced CE-SDS analysis indicated a time- and temperature-dependent increase in low-molecular-weight fragments and a corresponding decrease in the intact form, with more pronounced effects observed at 50 °C. Reduced CE-SDS revealed a more rapid increase in total impurity levels at 50 °C, accompanied by a decrease in total light and heavy chain content. SE-UPLC showed enhanced aggregation under thermal stress, more pronounced at 50 °C. LC-MS/MS analysis identified increased asparagine deamidation in the PENNY peptide and pyroglutamic acid formation (pE) at the N-terminus of the heavy chain. **Conclusions**: The degradation profiles of the biosimilar and originator mAbs were highly comparable under thermal stress, with no significant qualitative differences detected. By applying a multi-tiered analytical characterization technique, this study provides a comprehensive comparability assessment that underscores the robustness of biosimilarity even under forced degradation conditions.

## 1. Introduction

Capillary electrophoresis–sodium dodecyl sulfate (CE-SDS) is a widely used analytical technique in the biopharmaceutical industry for quantitative and qualitative assessment of size heterogeneity in therapeutic proteins [1]. Recently, CE-SDS has gained broad acceptance as a robust and standardized method for the characterization of monoclonal antibodies (mAb), particularly for evaluating their purity and impurity profiles [2,3,4,5,6]. Compared with traditional and labor-intensive SDS-polyacrylamide gel electrophoresis, CE-SDS offers superior resolution, reproducibility, automation, and robustness, making it the preferred method in many analytical workflows [7,8]. CE-SDS is typically performed under non-reducing (nrCE-SDS) and reducing (rCE-SDS) conditions to enable the separation and detection of product-related size variants, such as fragments and aggregates, in mAbs [9]. Under non-reducing conditions, the method primarily detects and quantifies intact antibodies, disulfide-linked low-molecular-weight (LMW) species, and covalently bound high-molecular-weight (HMW) species. In contrast, reducing conditions allow for the monitoring and quantification of free light (L) and heavy (H) chains, non-glycosylated heavy chain (NGH), and non-reducible species (NRS) [2,3,4]. These assays are well-established for evaluating mAb purity and are included in the United States Pharmacopoeia (USP) monograph <129> [5,10].

mAbs are the fastest-growing class of biotherapeutics and are extensively used to treat various diseases, including oncological, autoimmune, and cardiovascular diseases [11]. Patents for several originator (OR) biopharmaceutical products have either expired or are approaching expiration in the coming years. Therefore, biosimilar (BS) versions have been developed and manufactured by various biopharmaceutical companies over the past three decades [12,13,14]. BS products should demonstrate comparable quality, safety, and efficacy to their OR counterparts. To establish physicochemical and functional similarity, comprehensive analytical characterization is essential. Furthermore, accelerated and forced degradation (stress) studies are commonly conducted as part of biosimilarity assessments to evaluate their effects on product stability and overall quality [15,16]. mAbs are frequently exposed to various environmental stress conditions during manufacturing, storage, transportation, and administration. These stressors include agitation, light exposure, thermal stress, chemical oxidation, and freeze–thaw cycles, all of which can adversely affect their quality and potentially compromise therapeutic efficacy [15]. Among these, thermal stress is particularly versatile because it can induce fragmentation (resulting in LMW species) and aggregation (leading to HMW species) [15,16]. The formation of LMW and HMW species is considered a critical quality attribute (CQA) of mAbs and must be rigorously monitored throughout product development and manufacturing processes [17,18].

Thermal stress is commonly used as an accelerated degradation tool to evaluate mAb stability over shorter timeframes [19]. Previous studies have demonstrated that elevated temperatures promote fragmentation and/or aggregation, which ultimately reduces the concentration of functional antibodies [20,21]. CE-SDS is routinely used to analyze thermally induced mAb fragments because of its high-resolution separation of protein species based on molecular weight [22,23,24]. Vlasak et al. [25] emphasized the essential role of CE-SDS in monitoring mAb fragmentation, considering its implications for efficacy and safety. Furthermore, Dada et al. [26] reported that CE-SDS is a robust orthogonal method to size-exclusion chromatography (SEC) for detecting hinge region fragmentation in mAbs.

Comprehensive method validation is essential for ensuring the reliability, accuracy, and reproducibility of the nrCE-SDS and rCE-SDS methods for regulatory compliance and product quality assurance. Several previous studies have reported the validation of CE-SDS methods for assessing mAb purity [1,3,4,27,28,29]. In this study, we report the validation of CE-SDS methods for anti-VEGF mAb purity testing. Subsequently, the validated nrCE-SDS and rCE-SDS methods were applied to compare the fragmentation profiles of a BS product with those of its OR products sourced from the United States (OR-US) and the European Union (OR-EU) under thermal stress (37 °C and 50 °C) conditions for different time periods. The former temperature represents a physiologically relevant commonly applied in accelerated stability studies, while the latter serves as a more severe stress temperature condition, selected in line with regulatory guidance and remaining well below the thermal melting temperature of the anti-VEGF mAb. To gain a comprehensive understanding of the degradation behavior, degradation pathways were investigated using size-exclusion ultra-performance liquid chromatography (SE-UPLC) and peptide mapping by liquid chromatography–tandem mass spectrometry (LC–MS/MS) on samples subjected to 14 days of thermal stress. These analytical investigations are not only essential for comparability and regulatory assessment but also highly relevant from a clinical standpoint. Maintaining the stability of anti-VEGF mAb is of particular importance, as they are widely used in certain types of cancer and also treatment of retinal disorders, where any loss of stability during storage or transport may affect therapeutic efficacy and safety. In practice, strict cold-chain handling is required, and deviations from recommended conditions can increase the risk of degradation. Therefore, forced degradation studies provide not only regulatory and scientific insights but also important context for real-world handling and use of these therapeutics.

## 2. Results

### 2.1. Method Development and Validation of CE-SDS

First, robust nrCE-SDS and rCE-SDS methods were developed for monitoring purity and impurity levels using a single sample preparation with a single injection per replicate. The effects of sample concentration, incubation time, concentration of IAM (for non-reduced), and concentration of 2-mercaptoethanol (BME) (for reduced) were evaluated for method development. Section 4.3 describes the developed methods in detail. These methods were then validated according to the International Council for Harmonization of Technical Requirements for Pharmaceuticals for Human Use (ICH) Q2 (R2) guidelines by assessing the following parameters: specificity, linearity, accuracy, precision (repeatability and intermediate precision), limit of quantitation, range, and robustness [30]. Subsequently, the validated CE-SDS methods were applied to the thermally stressed mAb samples to evaluate their purity and impurity profiles. Table 1 presents a summary of the method validation results, and each validation parameter is described below.

#### 2.1.1. Specificity

The assay’s ability to resolve the product from potential impurities present in the blank injections (formulation buffer and SDS sample buffer) was evaluated to assess the method’s specificity. Single injections of individual preparations of the sample, formulation buffer, and SDS sample buffer were analyzed under both non-reducing and reducing conditions according to the analytical method. No peaks interfering with the analysis and integration of the sample were detected in the nrCE-SDS and rCE-SDS electropherograms of the formulation buffer or SDS sample buffer, indicating that the developed methods are specific for sample analysis (electropherograms not shown).

#### 2.1.2. Linearity

The linearity of the methods was evaluated by analyzing the sample in triplicate under both non-reducing and reducing conditions at five concentrations across the range of 5.0–15.0 mg/mL (5.0, 7.5, 10.0, 12.5, and 15.0 mg/mL). These concentrations correspond to 50–150% of the target sample concentration (10.0 mg/mL). For both the reducing and non-reducing conditions, the corrected peak areas for the observed peaks were plotted against the nominal sample concentration to assess linearity using regression analysis. The correlation coefficient (R^2^) was evaluated to confirm the linearity.

Under non-reducing conditions, mean corrected peak areas of intact IgG and total LMW species were plotted against nominal sample concentrations. The intact IgG and total LMW species exhibited acceptable linearity (R^2^ ≥ 0.95), with R^2^ values of 0.99 (Table 1). Under reducing conditions, the mean corrected peak areas of the L, H, and total impurities were plotted against the nominal sample concentration. As presented in Table 1, the L, H, and total impurities exhibited acceptable linearity (R^2^ ≥ 0.95), with R^2^ values of 0.99, 0.99, and 0.98, respectively. Both methods showed good linearity across the range of 50–150% of the target sample concentration.

#### 2.1.3. Accuracy

The accuracy of the method was demonstrated based on the results of both non-reducing and reducing sample preparations used in the linearity study. The predicted sample concentrations were calculated by applying the corrected peak areas of individual peaks to their respective calibration curves. The recovery percentages at each level of the linearity assessment were then determined by comparing the predicted concentrations with the nominal sample concentrations.

Under non-reducing conditions, the predicted concentrations of intact IgG and total LMW species were calculated using corrected peak areas and their respective linear regressions. As shown in Table 1, the recoveries for intact IgG and total LMW ranged from 90 to 116% and 91 to 128%, respectively. Under reducing conditions, the predicted concentrations of L, H, and total impurities were similarly calculated. The recovery values ranged from 87% to 109% for L, from 86% to 109% for H, and from 85% to 114% for the total impurity (Table 1). All results met the acceptance criteria of 70–130% for recovery.

#### 2.1.4. Precision (Repeatability and Intermediate Precision)

The precision of the methods was evaluated by performing repeatability and intermediate precision assays. To assess repeatability, the corrected peak areas obtained from the linearity study of triplicate preparations (non-reducing and reducing) at the target sample concentration of 10.0 mg/mL were analyzed. Under non-reducing conditions, the %RSD values for intact IgG and total LMW species were 2.0% and 1.8%, respectively (Table 1). Under reducing conditions, the %RSD values were 2.4% for L, 2.4% for H, and 4.5% for the total impurity (Table 1). These results meet the acceptance criterion of %RSD ≤ 5%.

The intermediate precision was assessed by two analysts using separate capillaries and sample gel buffers under both non-reducing and reducing conditions. The second analyst analyzed triplicate samples at a target concentration of 10.0 mg/mL using the analytical method. Under non-reducing conditions, the collective %RSD from both analysts (*n* = 6) for the corrected percent area of the intact IgG was 0.1% and 0.6% for the total LMW species (Table 1). Under reducing conditions, the collective %RSD from both analysts (*n* = 6) was 1.0% for L, 0.5% for H, and 2.2% for the total impurity (Table 1).

#### 2.1.5. Limit of Quantitation (LOQ)

The LOQ of the method was determined using consecutive analyses of samples with decreasing concentrations. Under both non-reducing and reducing conditions, the LOQ was calculated by dividing the area of an impurity peak by the total peak area obtained from a 10.0 mg/mL sample. The impurity peak was defined as any sample-related peak other than the intact IgG, exhibiting a signal-to-noise (S/N) ratio of approximately 10. The calculated LOQs for the percentage-corrected area were 0.8% under non-reducing conditions and 0.6% under reducing conditions (Table 1).

#### 2.1.6. Range

The ranges of the methods were determined based on the results obtained from the linearity, accuracy, precision, and LOQ evaluations and were found to be 1.25–15.0 mg/mL sample concentration under non-reducing conditions and 0.158–15.0 mg/mL under reducing conditions (Table 1).

#### 2.1.7. Robustness

The robustness of the nrCE-SDS and rCE-SDS methods was assessed using data from intermediate precision studies and under varying incubation times and concentrations of IAM under non-reducing conditions or BME under reducing conditions. Under non-reducing conditions, the %RSD values for intact IgG and total LMW species were 0.1% and 0.6%, respectively. Under reducing conditions, the %RSD values were 0.9% for L, 0.4% for H, and 2.5% for the total impurity (Table 1). These low %RSD values confirm the robustness of the CE-SDS assays under the tested conditions.

### 2.2. Comparison of Fragmentation Profiles at 37 °C

**Figure 1:**
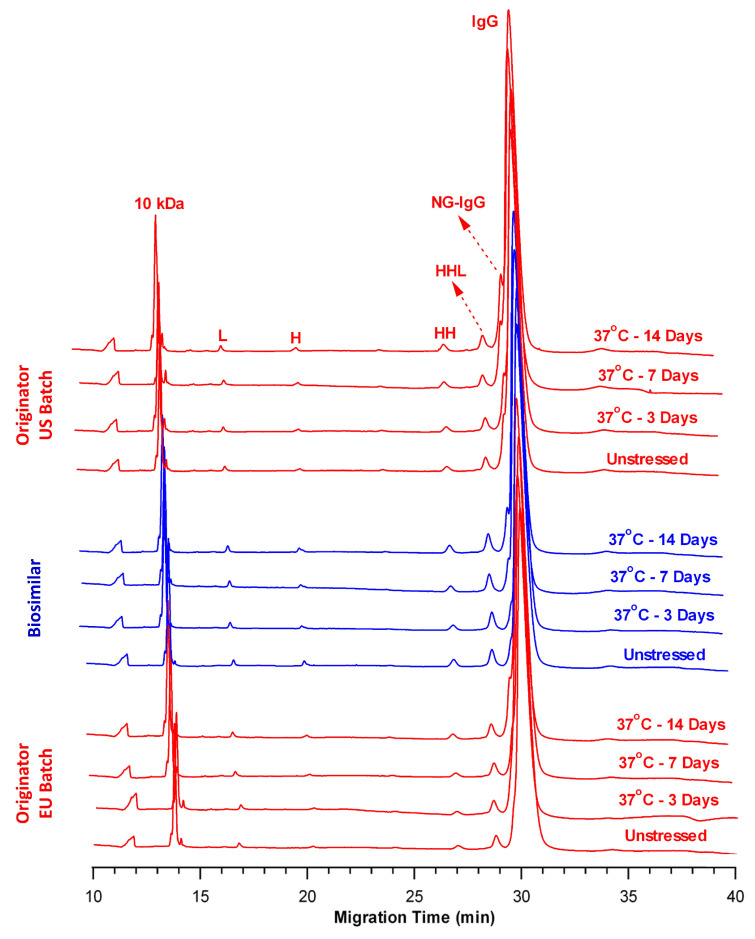
Overlay of nrCE-SDS electropherograms of the BS (blue) and OR (OR-US and OR-EU, red) products at 37 °C. (L: light chain, H: heavy chain, HH: heavy–heavy chain dimers, HHL: heavy–heavy–light chain trimers, NG-IgG: non-glycosylated IgG).

In the first part of the study, the samples (BS, OR-US, and OR-EU) were incubated at 37 °C for 3, 7, and 14 days to evaluate the formation and quantify the levels of degradation-induced fragment species. These assessments were performed using the validated nrCE-SDS and rCE-SDS methods. Figure 1 presents overlay nrCE-SDS electropherograms of the control and thermally stressed samples, where the profiles of OR-US and OR-EU are shown in red, and that of BS is presented in blue. Samples maintained under unstressed conditions served as controls throughout the study. In the nrCE-SDS analysis, species migrating before intact IgG, including the light chain (L, ~25 kDa), heavy chain (H, ~50 kDa), heavy–heavy chain dimers (HH, ~100 kDa), heavy–heavy–light chain trimers (HHL, ~125 kDa), and non-glycosylated IgG (NG-IgG, ~145 kDa), were categorized as LMW species and considered product-related impurities. The total area of these species was used as an indicator of fragmentation, whereas the intact IgG peak (~150 kDa) was regarded as the primary functional product. Peak assignments were based on the relative migration times of the species detected in the electropherogram compared with the standard IgG control.

Table 2 presents the relative percentages of LMW species and intact IgG for all samples across different time points, and they are expressed as means ± standard deviations (SDs) (n = 3). The total amount of LMW species exhibited a time-dependent increase from Day 3 to Day 14 across all samples (BS, OR-US, and OR-EU), as summarized in Table 2. In the control (unstressed) samples, the relative percentage of LMW species was 5.52% ± 0.05% for BS, 6.11% ± 0.05% for OR-US, and 5.34% ± 0.05% for OR-EU. Upon thermal stress exposure at 37 °C, the total LMW content in BS increased to 6.02% ± 0.02%, 7.21% ± 0.12%, and 8.80% ± 0.06% on Days 3, 7, and 14, respectively. Comparable trends were observed for the originator products, with OR-US showing increases to 7.26% ± 0.02%, 8.39% ± 0.12%, and 10.72% ± 0.02%, respectively, and OR-EU increasing to 6.10% ± 0.03%, 7.56% ± 0.10%, and 8.33% ± 0.04%, respectively, over the same time points. Figure 1 presents the gradual increase in total LMW content over the 14-day period. Among the individual impurity species, NG-IgG most significantly contributed to the observed increase. This increase is likely attributable to the cleavage of glycosidic bonds within the IgG molecule under thermal stress conditions at 37 °C, resulting in the formation of a non-glycosylated form. Simply, thermal stress can promote cleavage of N-linked glycosidic bonds between the asparagine side chain and the innermost N-acetylglucosamine (GlcNAc) residue of the Fc N-glycan. Cleavage of this glycosidic linkage leads to the loss of the entire N-glycan moiety, yielding an NG-IgG. The pronounced increase in NG-IgG relative to other impurity species under the tested stress conditions suggests that glycosidic bond cleavage at Asn303 is a dominant degradation pathway at 37 °C.

In contrast, the relative percentage of intact IgG decreased in all samples (BS, OR-US, and OR-EU) upon incubation at 37 °C (Table 2). For the BS sample, the intact IgG content decreased from 94.48% ± 0.04% in the control to 91.19% ± 0.05% after 14 days at 37 °C. A similar reduction was observed in the OR-US batch, with intact IgG decreasing from 93.89% ± 0.04% to 89.28% ± 0.02%, whereas in the OR-EU batch, levels declined from 94.66% ± 0.05% to 91.66% ± 0.04% over the same period. The overall reduction in intact IgG remained <5% for all samples throughout the 14-day incubation period. A comparison of the regression lines for intact IgG from the BS and OR products exhibited no significant difference in slope (*p* = 0.99). Collectively, the nrCE-SDS data indicated that the fragmentation profiles of the BS and both OR products exhibited comparable trends in response to thermal stress at 37 °C.

**Figure 2:**
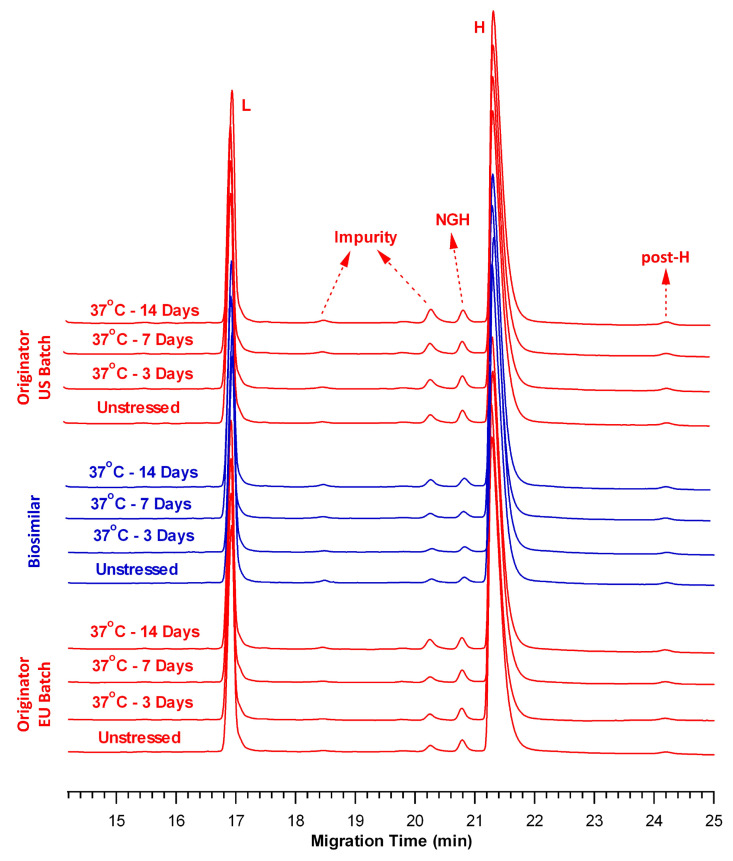
Overlay of rCE-SDS electropherograms of the BS (blue) and OR (OR-US and OR-EU, red) products at 37 °C. (L: light chain, H: heavy chain, NGH: non-glycosylated heavy chain, post-H: non-reducible mAb).

In the rCE-SDS assay, a predominant reduction in both inter- and intra-chain disulfide bonds enabled the quantification of free L chains, H chains, and NGH species. Figure 2 presents representative overlaid rCE-SDS electropherograms of the control and thermally stressed samples. Peaks observed between the L and H chains and those migrating after the H chain (post-H peaks) were classified as impurity species, and their total percentages were reported throughout the study [25]. The post-H peak indicates a non-reducible mAb form typically arising from incomplete reduction during sample preparation. In Figure 2, a very minor peak was observed at approximately 19.8 min, particularly after 14 days of incubation at 37 °C. However, the intensity of this peak was below the quantification threshold established by the validated CE-SDS method and was therefore excluded from the total impurity calculation for the 37 °C stress condition.

Table 3 presents the relative percentages of total impurities and total L + H content for the control and thermally stressed samples on Days 3, 7, and 14. In the control samples, the total impurity levels were 1.56% ± 0.03% for BS, 2.95% ± 0.10% for OR-US, and 2.21% ± 0.08% for OR-EU. Following incubation at 37 °C, impurity levels in BS increased to 1.85% ± 0.03%, 2.62% ± 0.02%, and 3.20% ± 0.08% on Days 3, 7, and 14, respectively. A comparable trend was observed for OR-US, with impurity levels increasing to 3.15% ± 0.04%, 3.36% ± 0.09%, and 4.03% ± 0.13%, and for OR-EU, with impurity levels increasing to 3.12% ± 0.03%, 3.19% ± 0.03%, and 3.98% ± 0.02% at the corresponding time points. Overall, Figure 2 shows that the fragmentation profiles of the BS and OR samples were similar under thermal stress conditions at 37 °C. Furthermore, the percentage of NGH species was consistently lower in the BS than in the OR, which may reflect differences in the cell line or manufacturing processes employed for each product.

In contrast, the total L + H content in the BS sample decreased from 98.44% ± 0.03% in the control to 96.80% ± 0.08% after 14 days of storage. A comparable reduction was observed in the OR-US batch, where the L + H content decreased from 97.05% ± 0.10% to 95.97% ± 0.13%. Similarly, the OR-EU batch exhibited a reduction from 97.79% ± 0.08% to 96.02% ± 0.02% over the same period (Table 3). The relative decreases in the L + H peak area were 1.64% for BS, 1.08% for OR-US, and 1.77% for OR-EU after 14 days at 37 °C. The slopes of the regression lines for the samples were statistically compared and exhibited a significant difference under reducing conditions at 37 °C (*p* < 0.05), which was attributed to the low level of total L + H in the control OR-US sample compared with the BS and OR-EU samples. Overall, the results of the rCE-SDS assay revealed that the changes in total impurity and L + H content followed nearly similar trends across all three products after 3, 7, and 14 days of storage at 37 °C, relative to their respective controls.

In addition to CE-SDS analysis, SE-UPLC and LC–MS/MS were employed to further investigate the degradation pathways of the samples subjected to 14 days of thermal stress. SE-UPLC analysis demonstrated an increase in total aggregate content (including dimers and multimers) in the BS from 2.11% (control) to 8.04% after incubation at 37 °C for 14 days. A similar trend was observed in OR-US and OR-EU products, where aggregate levels increased from 2.27% to 7.60% and from 2.72% to 7.11%, respectively. Conversely, the relative percentage of the monomer decreased in the BS, OR-US, and OR-EU samples at comparable percentages (ranging from 5.3% to 6.8%) following incubation at 37 °C. In the BS sample, the monomer content declined from 96.86% (control) to 90.26% after 14 days. A similar reduction was observed for OR-US, with monomer levels decreasing from 96.77% to 90.80%, while in OR-EU, the monomer content dropped from 96.26% to 91.30% over the same period. The comparative chromatograms of the samples are provided in the Supplementary File.

LC–MS/MS analysis revealed that two tryptic peptides have elevated levels of post-translational modifications (PTMs). The first PTM was the formation of pE through cyclization of the N-terminal glutamic acid (E) residue on the heavy chain HC:T1 tryptic peptide (amino acid sequence: EVQLVESGGGLVQPGGSLR). The second PTM was deamidation occurring on asparagine (N) residues on the heavy chain HC:T35 tryptic peptide (amino acid sequence: GFYPSDIAVEWESNGQPENNYK). The presence of PTMs was confirmed by analyzing the corresponding MS/MS fragment ions. Following thermal stress, pE levels in HC:T1 increased from 2.16% to 3.49% for BS, from 2.35% to 3.41% for OR-US, and from 3.03% to 3.94% for OR-EU. Likewise, the total deamidation of asparagine residues in HC:T35 increased from 3.87% to 6.67% for BS, from 4.32% to 7.05% for OR-US, and from 5.32% to 7.29% for OR-EU after incubation at 37 °C (the details of the peptide mapping analysis results are provided in the electronic Supplementary File).

### 2.3. Comparison of Fragmentation Profiles at 50 °C

**Figure 3:**
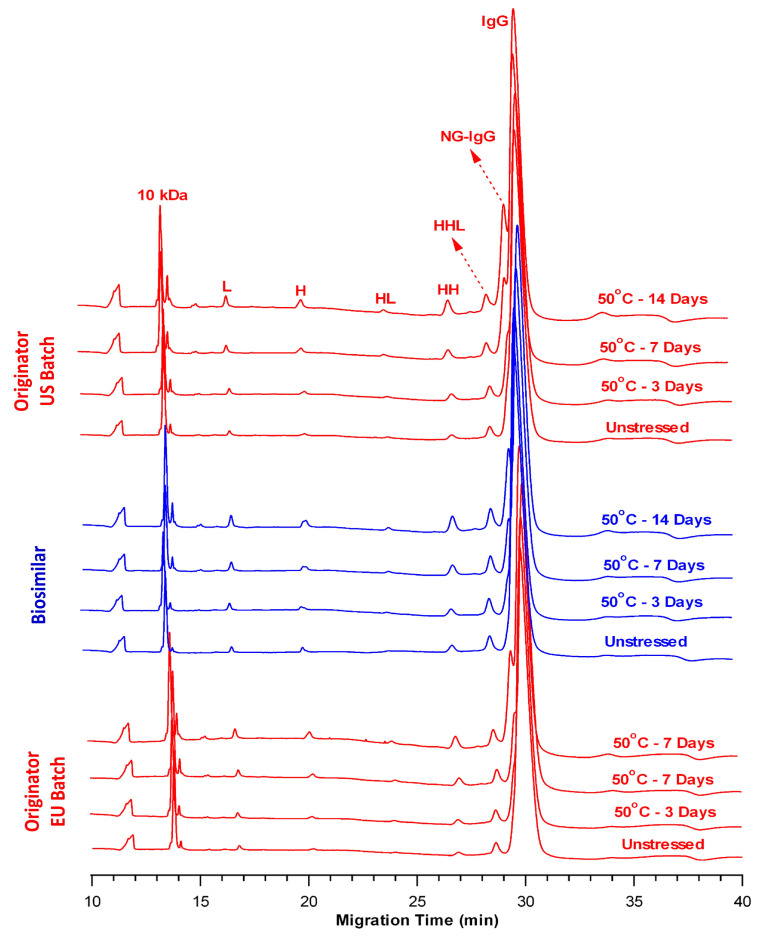
Overlay of nrCE-SDS electropherograms of the BS (blue) and OR (OR-US and OR-EU, red) samples at 50 °C. (L: light chain, H: heavy chain, HL: heavy–light chain dimers, HH: heavy–heavy chain dimers, HHL: heavy–heavy–light chain trimers, NG-IgG: non-glycosylated IgG).

To further investigate the effects of elevated temperature on the fragmentation behavior of the samples, the BS, OR-US, and OR-EU samples were incubated at 50 °C for 3, 7, and 10 days. Figure 3 presents an overlay of the nrCE-SDS electropherograms of the control and thermally stressed samples over the indicated time points (with BS shown in blue and OR-US and OR-EU shown in red).

As summarized in Table 4, the percentages of LMW species progressively increased with incubation time in all stressed samples relative to their respective controls. This increase was particularly evident on Days 7 and 14, with more pronounced peak intensities contributing to the LMW region (Figure 3). Quantitatively, the LMW content in BS increased from 5.52% ± 0.05% (control) to 19.71% ± 0.15% (Day 14). A similar trend was observed for the OR products: the LMW species increased from 6.11% ± 0.05% to 21.61% ± 0.13% for OR-US and from 5.34% ± 0.05% to 20.00% ± 0.02% for OR-EU (Table 4).

Concurrently, a time-dependent reduction in the percentage of intact IgG was observed across all samples. In BS, intact IgG levels decreased from 94.48% ± 0.04% (control) to 80.29% ± 0.15% (Day 14). Similarly, intact IgG content declined from 93.89% ± 0.04% to 78.39% ± 0.13% in OR-US and from 94.66% ± 0.05% to 80.00% ± 0.02% in OR-EU over the same period. These findings demonstrate that thermal stress at 50 °C for 14 days resulted in a substantial reduction (~20%) in mAb purity due to increased fragmentation. The statistical analysis revealed that the degradation rates of intact IgG at 50 °C were comparable between the products (*p* = 0.066). The nrCE-SDS data consistently exhibited comparable fragmentation patterns among the BS and OR batches, as revealed by the increased number of LMW species and reduced intact IgG levels.

**Figure 4:**
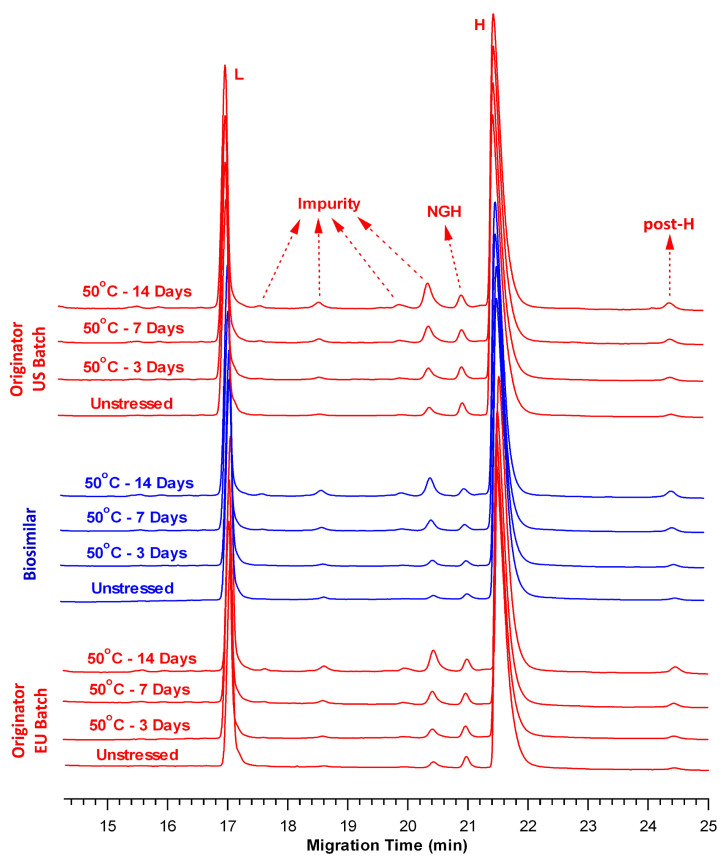
Overlay of rCE-SDS electropherograms of the BS (blue) and OR (OR-US and OR-EU, red) products at 50 °C. (L: light chain, H: heavy chain, NGH: non-glycosylated heavy chain, post-H: non-reducible mAb).

Under reducing conditions, the electropherograms of the BS and OR batches exhibited comparable fragmentation profiles at 50 °C (Figure 4). Relative to the respective controls, the rCE-SDS profiles exhibited a progressive increase in the impurity peaks and a concomitant decrease in the total L + H content over the incubation period. After 14 days at 50 °C, the total impurity content in the BS sample increased from 1.56% ± 0.03% to 5.56% ± 0.13% (Table 5). Similarly, the impurity levels increased from 2.95% ± 0.10% to 8.06% ± 0.05% for OR-US and from 2.21% ± 0.08% to 7.09% ± 0.09% for OR-EU.

In contrast, the total L + H content exhibited a time-dependent decline across all samples (Table 5). In particular, BS exhibited a reduction from 98.44% ± 0.03% (control) to 94.44% ± 0.13% (Day 14). A similar trend was observed for OR-US (from 97.05% ± 0.10% to 91.94% ± 0.05%) and OR-EU (from 97.79% ± 0.08% to 92.91% ± 0.09%) over the same period. The slopes of the regression lines were statistically compared and indicated comparable degradation rates (*p* = 0.066). Collectively, the results obtained from both nrCE-SDS and rCE-SDS analyses indicate that thermal stress at 50 °C induces comparable fragmentation profiles in BS and OR batches, characterized by increased impurity formation and decreased levels of total L + H content.

SE-UPLC analysis revealed a substantial increase in total aggregate content in the BS, rising from 2.11% (control) to 20.17% after 14 days of incubation at 50 °C, representing approximately a 9.6-fold increase. A comparable trend was observed in the OR products, with aggregate levels increasing from 2.27% to 21.65% (~9.5-fold) for OR-US and from 2.72% to 19.32% (~7.1-fold) for OR-EU. Conversely, the relative monomer content showed a marked decrease across all samples. In the BS, the monomer level declined from 96.86% to 76.93%, representing a relative decrease of approximately 20.6%. Similarly, monomer levels dropped from 96.77% to 75.35% in OR-US (~22.1% decrease) and from 96.38% to 77.93% in OR-EU (~19.1% decrease) over the same period at 50 °C. The comparative chromatograms of the samples are provided in the Supplementary File.

LC–MS/MS analysis demonstrated that pE formation in the heavy chain tryptic peptide (HC:T1) increased significantly following thermal stress. Specifically, pE levels increased from 2.16% to 10.86% in the BS, from 2.35% to 10.22% in OR-US, and from 3.03% to 11.56% in OR-EU after 14 days of incubation at 50 °C. Similarly, total deamidation of asparagine residues in the HC:T35 tryptic peptide increased from 3.87% to 11.55% in BS, from 4.32% to 11.46% in OR-US, and from 5.32% to 11.73% in OR-EU under the same stress conditions (the details of the peptide mapping analysis results are provided in the electronic Supplementary File).

## 3. Discussion

Forced degradation studies are essential for elucidating the potential degradation pathways of mAbs under various stress conditions, including light exposure, temperature, pH changes, and freeze–thaw cycles [16]. Among these, thermal stress is a commonly applied approach for evaluating the extent of degradation. mAbs are large and structurally complex biopharmaceuticals and are typically stored at 5 °C ± 3 °C. They exhibit high sensitivity to elevated temperatures, which can lead to global structural disruption through both physical and chemical degradation mechanisms [21,31,32]. The predominant degradation pathways under thermal stress are fragmentation (resulting in the formation of LMW species) and aggregation (resulting in the formation of HMW species). These product-related impurities are considered CQAs and require careful monitoring throughout the product’s life cycle [33].

CE-SDS is a preferred analytical technique for mAb purity assessment and size variant profiling, particularly for detecting LMW fragment species. In this study, a comprehensive comparative analysis of the BS and OR batches was performed to evaluate the fragmentation profiles of the products under two elevated temperature conditions (37 °C and 50 °C) over various time periods. These temperature points were selected based on regulatory guidance and the biophysical properties of the tested mAbs. Incubation at 37 °C is a physiologically relevant condition and is commonly used in accelerated stability studies. In contrast, 50 °C was selected as a more severe stress condition, meeting the criterion of being at least 10 °C above the standard accelerated testing temperature. Importantly, both temperature conditions remain well below the thermal melting temperature (Tm) range of the anti-VEGF mAb (72.5–73.1 °C) [34], thereby ensuring that the product’s structural integrity was not entirely compromised during the incubation period.

An overlay of nrCE-SDS electropherograms exhibits degradation in the form of LMW species across all samples (Figure 1 and Figure 3). These fragments increased consistently, with thermal stress at 50 °C inducing a more pronounced increase than thermal stress at 37 °C. After 14 days at 50 °C, BS exhibited a level of LMW species (19.71% ± 0.15%) comparable to that of the OR-US (21.61% ± 0.13%) and OR-EU (20.00% ± 0.02%) (Table 4). The primary contributor to the LMW species is the NG-IgG form, which is likely generated by hydrolysis of the mAb under slightly acidic conditions (formulation buffer pH 6.2) during prolonged exposure to elevated temperature. Correspondingly, a significant decrease in the percentage of intact IgG was observed under thermal stress in all samples, as assessed by nrCE-SDS. At 37 °C, the purity of intact IgG ranged from 89% to 92%. As expected, a greater decrease in intact IgG was observed at 50 °C. In particular, the level of intact IgG in BS samples decreased to 80.29% ± 0.15%, whereas comparable reductions were observed in OR-US (78.39% ± 0.13%) and OR-EU (80.00% ± 0.02%) (Table 4). Collectively, these results indicate that thermal stress promotes mAb truncation, generating fragments with molecular weights below that of the full antibody (~150 kDa). Overall, the fragmentation profiles of the mAb samples were similar throughout the thermal stress study.

In contrast, the cumulative L + H content gradually decreased at both 37 °C and 50 °C, with the latter condition exerting a more pronounced effect (Table 3 and Table 5). Comparative rCE-SDS electropherograms of the control and thermally stressed BS and OR samples revealed an increasing trend in the total impurities over time (Figure 2 and Figure 4) under both temperature conditions. After 14 days at 50 °C, the BS sample exhibited a lower level of total impurities (5.56% ± 0.13%) than OR-US (8.06% ± 0.05%) and OR-EU (7.09% ± 0.09%). Furthermore, our data indicated that BS contained a slightly lower amount of NGH than the OR batches, suggesting a higher degree of glycan occupancy at the conserved asparagine (Asn) site. Differences in NGH levels for BS mAbs have been previously reported by several research groups [34,35,36,37]. The observed variation in impurity levels may be attributed to differences in the cell lines and manufacturing processes used for BS and OR production [37]. NGH is generally regarded as a CQA with minimal clinical impact; however, its presence may have potential functional implications. Glycosylation of the Fc region plays a critical role in maintaining proper protein folding, structural integrity, and effector functions. A lack of glycosylation may lead to reduced thermal stability and altered conformational dynamics of mAbs. NGH typically does not significantly affect the overall potency or safety profile of therapeutic antibodies; however, monitoring its levels remains important for quality and comparability assessments [34,35,36,37].

Furthermore, the post-H peak level, as shown in the rCE-SDS electropherograms, slightly increased in all samples at 50 °C over time (Figure 4). In contrast, the increase was negligible at 37 °C over the same period (Figure 2). This observation may be explained by the formation of thioether-linked antibody-fragment species at elevated temperatures [38]. Moreover, the peaks observed between L and NGH correspond to the cleaved forms of the mAb’s structure, which are more readily detectable under thermal stress [25]. Overall, the rCE-SDS profiles (Figure 2 and Figure 4) of the BS and OR batches exhibited similar qualitative fragmentation patterns under thermal stress conditions, with no distinct or product-specific peaks identified in any sample.

Both the nrCE-SDS and rCE-SDS results revealed that the fragmentation profiles of the BS and OR batches were highly similar under the same thermal stress conditions. From a practical perspective, compared with thermal stress at 37 °C, thermal stress at 50 °C led to an increased formation of impurity peaks, accompanied by a concomitant reduction in purity peaks.

Moreover, the results obtained from SE-UPLC and LC–MS/MS provided deeper insights into the degradation pathways of the BS, OR-US, and OR-EU products. Specifically, thermal stress is a well-established factor that promotes the formation of aggregates and PTMs in mAbs. Elevated temperatures can destabilize the native conformation of mAbs, resulting in partial unfolding and subsequent aggregation. These aggregates can adversely affect product stability, increase immunogenic potential, and reduce therapeutic efficacy [39]. SE-UPLC is widely used for the sensitive detection and quantification of mAb aggregates. In this study, exposure to thermal stress conditions (37 °C and 50 °C) led to a marked increase in the total aggregate content, as detected by SE-UPLC, highlighting temperature-induced aggregation as a key degradation pathway for the tested samples. Notably, the absence of aggregate species in the CE-SDS analysis suggests that the aggregates detected by SE-UPLC are predominantly non-covalent in nature, which is consistent with a previous report [40]. Moreover, the CE-SDS method used in this study involves a denaturing separation environment, including SDS in the background electrolyte solution (BGE), and non-covalent aggregates are dissociated and therefore not detectable under these conditions.

In addition to aggregation induced by thermal stress, mAbs containing an N-terminal glutamic acid (E) residue are susceptible to cyclization, resulting in the formation of pyroglutamic acid (pE). This modification can affect product stability by altering the charge profile and structural integrity of the antibody [41]. The formation of pE is a well-characterized, temperature-dependent, non-enzymatic modification, with elevated stress conditions accelerating the conversion rate. In our study, 14 days of thermal incubation led to a measurable increase in the relative abundance of pE on the HC:T1 tryptic peptide, indicating that N-terminal cyclization is enhanced at elevated temperatures. Chelius et al. [41] demonstrate that the rate of pE formation increases with temperature, reporting 4.5% and 7.0% at 37 °C and 45 °C, respectively. Our results are consistent with these findings, showing approximately 3.5% and 10% at 37 °C and 50 °C, despite differences in the mAbs studied. Furthermore, deamidation of N residues is a well-known non-enzymatic PTM and a major chemical degradation pathway in mAbs, particularly under thermal stress. This modification can compromise the stability and shelf life of therapeutic antibodies [42]. Notably, the presence of the PENNY sequence motif within the Fc region renders this site especially susceptible to deamidation [43]. Following 14 days of thermal stress, the total deamidation level on the PENNY peptide—defined as the sum of modifications at N390, N395, and N396 residues—was comparable among the BS, OR-US, and OR-EU samples. With respect to deamidation, the levels observed in our study for control samples are nearly 3%, in good agreement with the unstressed samples reported by Saleem et al. [37]. Importantly, Chelius et al. [43] also reported that deamidation rates increase under accelerated conditions, which is consistent with the elevated levels we observed in our stressed samples.

In our work, each analytical technique provided complementary information for biosimilarity assessment. CE-SDS enabled the evaluation of size variants at the intact protein level, offering robust quantitative assessment of purity, molecular integrity, and potential degradation products under both reducing and non-reducing conditions. SE-UPLC complemented this by detecting and quantifying soluble aggregates and fragments, thereby providing critical insights into size heterogeneity. LC–MS/MS-based peptide mapping enabled high-resolution characterization at the primary structure level, allowing the identification and localization of PTMs such as deamidation and pyroglutamic acid formation. Together, these orthogonal methods covered multiple structural levels of characterization, ensuring a comprehensive comparability exercise. This multi-tiered approach strengthens biosimilarity assessment by mitigating the limitations of any single method and enabling a more complete evaluation of quality attributes.

## 4. Materials and Methods

### 4.1. Materials and Reagents

Both the BS and OR mAbs (anti-VEGF, bevacizumab is the active substance) are humanized monoclonal IgG1 antibodies produced using recombinant DNA technology in a Chinese hamster ovary (CHO) mammalian cell line. The BS product was developed and manufactured by Turgut Ilaclari A.S. (Istanbul, Türkiye) as a proposed BS product to the OR product containing the same active substance. Two OR batches (brand name: Avastin, Genentech/Roche), one from the United States [U.S.] and one from the European Union [EU], were procured and handled according to the manufacturer’s instructions. The concentrations and compositions of the formulation buffer (i.e., buffering agents and surfactants) were identical for both the BS and OR products.

All chemicals and reagents used in this study were of analytical grade, unless otherwise stated. Iodoacetamide (IAM), dithiothreitol (DTT), guanidine hydrochloride, ammonium bicarbonate, monosodium phosphate, sodium iodide, and mineral oil were purchased from Sigma-Aldrich (St. Louis, MO, USA), and BME was obtained from Bio-Rad (Hercules, CA, USA). A 10 kDa internal standard, IgG control standard, acidic wash solution (0.1 M hydrochloric acid), basic wash solution (0.1 M sodium hydroxide), SDS-gel buffer, SDS sample buffer, and a bare-fused silica capillary were purchased from AB Sciex LLC (Framingham, MA, USA). Sodium chloride, trifluoracetic acid (TFA), and acetonitrile were obtained from Merck (Darmstadt, Germany). *Rapi*Gest^®^ SF and leucine enkephalin were purchased from Waters (Milford, MA, USA). Trypsin (sequencing grade) was provided by Promega (Madison, WI, USA). Sample vials and caps for CE-SDS analysis were obtained from AB Sciex (Framingham, MA, USA). Ultrapure water (18.2 MΩ.cm) was prepared in-house using a Milli-Q Direct 8 system (Merck Millipore, Darmstadt, Germany).

### 4.2. Preparation of Thermally Stressed Samples

Thermal stress experiments were performed using a ThermoMixer^®^ C thermoblock (Eppendorf, Hamburg, Germany) by placing the sample-containing tubes vertically away from direct light. Then, 200 µL of sample aliquots was transferred into separate 0.5 mL Eppendorf tubes (Eppendorf, Hamburg, Germany) and incubated for 3, 7, and 14 days at 37 °C or 50 °C without orbital shaking. The withdrawn stressed samples were analyzed using both nrCE-SDS and rCE-SDS assays. A triple-sample preparation was performed, followed by a single injection per preparation, with the mean of three consecutive runs reported. The stressed samples were prepared side by side for a comparability study, and their corresponding control samples were also analyzed.

### 4.3. CE-SDS

The CE-SDS experiments were performed on a PA800 Plus Pharmaceutical Analysis System (Beckman Coulter, Brea, CA, USA) equipped with a UV diode-array detector set to 220 nm. All separations were performed using a bare-fused-silica capillary (50 µm ID, 360 µm OD) with a total length of 30.2 cm and a detection window located 20 cm from the inlet. The capillary cartridge was maintained at 20 °C throughout the analysis. A 10 kDa internal standard was used to normalize the migration time of the peaks. The IgG control standard was prepared and analyzed using nrCE-SDS and rCE-SDS as a system suitability assay before analysis of the stress samples.

The CE-SDS method was conducted in accordance with USP <129> parameters, with minor adjustments made to optimize the performance for the tested mAb samples [10]. Under non-reducing conditions, the samples were diluted to a final concentration of 10 mg/mL with Milli-Q water. A 10 µL aliquot of this solution was mixed with 90 µL of non-reducing sample buffer (NRSB) and 2 µL of a 10 kDa internal standard. The NRSB comprised 50 µL of 250 mM IAM and 750 µL of SDS sample buffer. The total mixture was vortexed thoroughly, centrifuged at 14,000 rpm for 30 s, and then heated at 70 °C for 10 min. After cooling for 3 min, the mixture was vortexed again, centrifuged at 10,000 rpm for 5 min to remove air bubbles, and transferred to a sample vial for CE-SDS analysis. Under reducing conditions, a reducing sample buffer (RSB) was used, comprising 50 µL of BME and 750 µL of SDS sample buffer. A 10 µL aliquot of this solution was mixed with 90 µL of RSB, followed by the addition of 2 µL of the 10 kDa internal standard. The mixture was vortexed, centrifuged at 14,000 rpm for 30 s, and then heated at 70 °C for 10 min. After cooling for 3 min, the mixture was vortexed again, centrifuged at 10,000 rpm for 5 min, and finally transferred to a sample vial for analysis. To prevent the evaporation of water during analysis, the tops of the gel mixture and sample buffer-containing vials were covered with a thin layer of mineral oil.

After CE separation between each run, the capillary was rinsed and preconditioned with a basic wash solution (for 10 min at 20 psi), an acidic wash solution (for 5 min at 20 psi), ultrapure water (for 2 min at 20 psi), and an SDS-gel buffer (for 10 min at 70 psi). The analytes were separated following initial capillary rinsing with a basic solution (for 3 min at 70 psi), an acidic solution (for 1 min at 70 psi), ultrapure water (for 1 min at 70 psi), and an SDS-gel buffer (for 10 min at 70 psi). The samples were then electrokinetically injected into the capillary at 5 kV for 30 s and separated at 15 kV in reverse polarity for 40 min under non-reducing and reducing conditions. Data were acquired at a sampling rate of 2 Hz. The sample tray was maintained at 10 °C during analysis. System control and data acquisition were performed using 32 Karat^TM^ software (v10.1, Beckman Coulter, Brea, CA, USA), and data were then exported to Empower^TM^ 3 software (Waters, Milford, MA, USA). Data processing and calculation of the percent time-corrected area (%TCA) for each species were performed using Empower^TM^ 3 software. The corrected peak area for each fragment was obtained by normalizing the individual peak area to the total peak area of all relevant species in the electropherogram.

Statistical analyses were performed using GraphPad Prism v9.1.3 (San Diego, CA, USA). To compare the degradation rates, *p*-values were calculated based on the slopes of the linear regression lines generated from the BS and OR sample groups. Results are expressed as means ± standard deviations (SDs), with a significance level set at *p* < 0.05.

### 4.4. SE-UPLC

Size-exclusion ultra-performance liquid chromatography (SE-UPLC) was conducted using an ACQUITY UPLC^®^ H-Class Bio system equipped with a titanium flow cell (Waters, Milford, MA, USA). Separation was performed on an ACQUITY UPLC^®^ Protein BEH SEC200 column (200 Å, 1.7 µm, 4.6 × 300 mm; Waters, Milford, MA, USA). The mobile phase consisted of 20 mM sodium phosphate and 188 mM sodium chloride, adjusted to pH 7.4. Chromatographic separation was carried out under isocratic conditions at a flow rate of 0.25 mL/min, with the column maintained at 30 °C. Samples were diluted to a final concentration of 1.25 mg/mL in the mobile phase, and 5 µL was injected per run. Eluting species were monitored by UV detection at 280 nm. Instrument control, data acquisition, and processing were performed using Empower^TM^ 3 software (Waters, Milford, MA, USA).

### 4.5. LC-MS/MS

Liquid chromatography–tandem mass spectrometry (LC–MS/MS) analysis was performed using an ultra-performance liquid chromatography system (ACQUITY UPLC^®^ H-Class Bio) coupled online to a Xevo G2–XS QTOF hybrid mass spectrometer (Waters, Milford, MA, USA). The mass spectrometer was equipped with an electrospray ionization (ESI) source operating in positive ion mode, employing MS^E^ functionality over an *m/z* range of 50–2000. The capillary and cone voltages were set to 1000 V and 25 V, respectively. Source and desolvation temperatures were maintained at 100 °C and 350 °C, respectively. Sampling cone and desolvation gas flows were set to 50 L/h and 800 L/h, respectively. The autosampler temperature was kept at 10 °C. External calibration was performed prior to analysis using a sodium iodide solution (2 μg/μL). Leucine enkephalin (*m/z* 556.2766) was continuously infused during data acquisition to serve as a lock mass for correction of mass accuracy drift. The instrument control, data acquisition, and data processing were carried out using UNIFI^TM^ (v1.9.4) Scientific Information System software.

Sample preparation involved denaturation of 100 μg of protein with 8 M guanidine hydrochloride, followed by reduction with dithiothreitol (DTT) at 57 °C for 30 min. Reduced cysteine residues were alkylated using iodoacetamide in the dark at room temperature for 30 min. Subsequently, buffer exchange into 50 mM ammonium bicarbonate (pH 8.0) was performed using polyacrylamide desalting columns to prepare the samples for enzymatic digestion. *Rapi*Gest™ SF surfactant was added to a final concentration of 0.1%, and trypsin was introduced at a 1:25 (*w*/*w*) enzyme-to-substrate ratio. Digestion proceeded for 1 h at 37 °C and was terminated by acidification with 50% trifluoroacetic acid (TFA). Following digestion, insoluble material was removed by centrifugation at 10 °C. The clarified supernatant was transferred to autosampler vials, and 45 μL aliquots were injected for analysis. Chromatographic separation was achieved with a 90 min run time, including a 70 min linear gradient from 5% to 60% acetonitrile. Mobile phases consisted of ultrapure water (A), 100% acetonitrile (B), and 1% trifluoroacetic acid (C). The column temperature was maintained at 40 °C. Eluted peptides were detected by UV absorbance at 214 nm prior to mass spectrometric analysis. Carbamidomethylation of cysteine residues was set as a fixed modification during data processing. Peptide identification was based on accurate mass measurements from MS data, comparing observed ions with theoretically calculated masses from in silico proteolytic digestion of the target protein. Peptide fragmentation produced *b*- and *y*-type ions, which facilitated amino acid sequence confirmation. PTM localization is performed by integrating accurate mass measurements with fragment ion analysis. For each modified peptide, UNIFI^TM^ evaluates the observed fragment ions, and by determining which ions incorporate the mass shift, the software assigns the modification to a specific amino acid residue. At least three fragment ions from MS/MS spectra were required for peptide sequence verification. One missed cleavage was permitted in peptide mapping analyses. Sequence confirmation was accepted with precursor ion mass accuracy within ±8 ppm.

## 5. Conclusions

In this study, we evaluated the comparability of the degradation profiles between the BS and OR mAb products under thermal stress conditions at 37 °C and 50 °C over incubation periods of 3, 7, and 14 days. The fragmentation levels were monitored using validated CE-SDS methods after exposure to thermal stress. The results revealed that thermal stress-induced fragmentation occurred in all mAb samples under study. Comparative nrCE-SDS and rCE-SDS analyses revealed that the BS, OR-US, and OR-EU products exhibited highly similar fragmentation profiles, characterized by comparable types and levels of LMW species across all tested conditions. A greater fragmentation extent was observed at 50 °C than at 37 °C, with degradation becoming particularly pronounced after 14 days, resulting in approximately 20% structural breakdown of the antibody. The degradation pathways and trends were consistent across all samples, time points, and temperatures. Furthermore, the validated CE-SDS methods exhibited robust performance in monitoring thermal stress-induced fragmentation and are suitable for both routine quality assessment and forced degradation studies. Additionally, the degradation pathway involves aggregate and PTM formations, as evidenced by comparable results from SE-UPLC and LC-MS/MS analyses of thermally stressed samples after 14 days. Overall results demonstrate that forced degradation studies provide valuable insights into the degradation pathways, supporting the development and comparability assessment of biosimilar projects.

## Figures and Tables

**Table 1:** Summary of CE-SDS method validation.

Validation Parameters		nrCE-SDS		rCE-SDS		
		Intact IgG	Total LMW	L	H	Total Impurity
**Specificity**		No interference		No interference		
**Linearity**		R^2^ = 0.99	R^2^ = 0.99	R^2^ = 0.99	R^2^ = 0.99	R^2^ = 0.98
**Accuracy**		90–116%	91–128%	87–109%	86–109%	85–114%
**Precision**	**Repeatability**	RSD = 2.0%	RSD = 1.8%	RSD = 2.4%	RSD = 2.4%	RSD = 4.5%
	**Intermediate Precision**	RSD = 0.1%	RSD = 0.6%	RSD = 1.0%	RSD = 0.5%	RSD = 2.2%
**Limit of Quantitation**		0.8%		0.6%		
**Range**		1.25–15.0 mg/mL		0.158–15.0 mg/mL		
**Robustness**		Complies				

**Table 2:** nrCE-SDS results of the control and thermally stressed samples at 37 °C. Results are presented as area% and means ± standard deviations (SDs) (n = 3).

Sample Name	Time Point	Total LMW (%)	Intact IgG (%)
**OR-US**	Control	6.11 ± 0.05	93.89 ± 0.04
	Day 3	7.26 ± 0.02	92.73 ± 0.02
	Day 7	8.39 ± 0.12	91.61 ± 0.13
	Day 14	10.72 ± 0.02	89.28 ± 0.02
**BS**	Control	5.52 ± 0.05	94.48 ± 0.04
	Day 3	6.02 ± 0.02	93.98 ± 0.02
	Day 7	7.21 ± 0.12	92.79 ± 0.12
	Day 14	8.80 ± 0.06	91.19 ± 0.05
**OR-EU**	Control	5.34 ± 0.05	94.66 ± 0.05
	Day 3	6.10 ± 0.03	93.90 ± 0.02
	Day 7	7.56 ± 0.10	92.44 ± 0.10
	Day 14	8.33 ± 0.04	91.66 ± 0.04

**Table 3:** rCE-SDS results of the control and thermally stressed samples at 37 °C. Results are presented as area% and means ± standard deviations (SDs) (n = 3).

Sample Name	Time Point	Total Impurity (%)	L + H (%)
**OR-US**	Control	2.95 ± 0.10	97.05 ± 0.10
	Day 3	3.15 ± 0.04	96.85 ± 0.04
	Day 7	3.36 ± 0.09	96.64 ± 0.09
	Day 14	4.03 ± 0.13	95.97 ± 0.13
**BS**	Control	1.56 ± 0.03	98.44 ± 0.03
	Day 3	1.85 ± 0.03	98.15 ± 0.03
	Day 7	2.62 ± 0.02	97.38 ± 0.02
	Day 14	3.20 ± 0.08	96.80 ± 0.08
**OR-EU**	Control	2.21 ± 0.08	97.79 ± 0.08
	Day 3	3.12 ± 0.03	96.88 ± 0.03
	Day 7	3.19 ± 0.03	96.81 ± 0.03
	Day 14	3.98 ± 0.02	96.02 ± 0.02

**Table 4:** nrCE-SDS results of the control and thermally stressed samples at 50 °C. Results are presented as area% and means ± standard deviations (SDs) (n = 3).

Sample Name	Time Point	Total LMW (%)	Intact IgG (%)
**OR-US**	Control	6.11 ± 0.05	93.89 ± 0.04
	Day 3	9.65 ± 0.03	90.35 ± 0.03
	Day 7	14.17 ± 0.02	85.83 ± 0.02
	Day 14	21.61 ± 0.13	78.39 ± 0.13
**BS**	Control	5.52 ± 0.05	94.48 ± 0.04
	Day 3	9.40 ± 0.05	90.60 ± 0.05
	Day 7	13.75 ± 0.11	86.25 ± 0.11
	Day 14	19.71 ± 0.15	80.29 ± 0.15
**OR-EU**	Control	5.34 ± 0.05	94.66 ± 0.05
	Day 3	9.18 ± 0.05	90.82 ± 0.05
	Day 7	13.61 ± 0.03	86.39 ± 0.03
	Day 14	20.00 ± 0.02	80.00 ± 0.02

**Table 5:** rCE-SDS results of the control and thermally stressed samples at 50 °C. Results are presented as area% and means ± standard deviations (SDs) (n = 3).

Sample Name	Time Point	Total Impurity (%)	L + H (%)
**OR-US**	Control	2.95 ± 0.10	97.05 ± 0.10
	Day 3	4.88 ± 0.06	95.12 ± 0.06
	Day 7	6.41 ± 0.10	93.59 ± 0.10
	Day 14	8.06 ± 0.05	91.94 ± 0.05
**BS**	Control	1.56 ± 0.03	98.44 ± 0.03
	Day 3	2.95 ± 0.06	97.05 ± 0.06
	Day 7	4.52 ± 0.10	95.48 ± 0.10
	Day 14	5.56 ± 0.13	94.44 ± 0.13
**OR-EU**	Control	2.21 ± 0.08	97.79 ± 0.08
	Day 3	4.03 ± 0.02	95.97 ± 0.02
	Day 7	5.83 ± 0.08	94.17 ± 0.08
	Day 14	7.09 ± 0.09	92.91 ± 0.09

## Data Availability

The data presented in this study are included in the article.

## Supplementary Materials

The following supporting information can be downloaded at https://www.mdpi.com/article/10.3390/ph18091267/s1. The comparative SE-UPLC chromatograms and tandem mass spectrometry data for samples are presented in the Supplementary File. Figure S1: Overlay of SE-UPLC Chromatograms of OR-US Sample under Thermal Stress. Figure S2: Overlay of SE-UPLC Chromatograms of BS Sample under Thermal Stress. Figure S3: Overlay of SE-UPLC Chromatograms of OR-EU Sample under Thermal Stress. Table S1: Comparison of Expected and Observed Masses for Modified HC:T1 Peptide in Samples Incubated at 37 °C. Modification site is shown in bold red font. Table S2: Comparison of Expected and Observed Masses for Modified HC:T1 Peptide in Samples Incubated at 50 °C. Modification site is shown in bold red font. Table S3: Comparison of Expected and Observed Masses for Modified HC:T39 Peptide in Samples Incubated at 37 °C. Modification sites are shown in bold red font. Table S4: Comparison of Expected and Observed Masses for Modified HC:T39 Peptide in Samples Incubated at 50 °C. Modification sites are shown in bold red font.

## Abbreviations

The following abbreviations are used in this manuscript:

BGE	Background Electrolyte Solution
BME	2-mercaptoethanol
BS	Biosimilar
CE-SDS	Capillary Electrophoresis–Sodium Dodecyl Sulfate
CHO	Chinese Hamster Ovary
CQA	Critical Quality Attribute
DTT	Dithiothreitol
E	Glutamic Acid
H	Heavy Chain
HHL	Heavy–Heavy–Light Chain
HMW	High Molecular Weight
IAM	Iodoacetamide
ICH	International Council for Harmonization
L	Light Chain
LC-MS/MS	Liquid Chromatography–Tandem Mass Spectrometry
LMW	Low Molecular Weight
LOQ	Limit of Quantitation
mAb	Monoclonal Antibody
N	Asparagine
NGH	Non-Glycosylated Heavy Chain
NG-IgG	Non-Glycosylated Immunoglobulin G
nrCE-SDS	Non-reducing Capillary Electrophoresis of Sodium Dodecyl Sulfate
NRS	Non-reducible species
OR	Originator
OR-EU	Originator-European Union
OR-US	Originator-United States
R^2^	Correlation Coefficient
rCE-SDS	Reducing Capillary Electrophoresis of Sodium Dodecyl Sulfate
RSB	Reducing Sample Buffer
RSD	Relative Standard Deviation
S/N	Signal-to-Noise
SD	Standard Deviation
SE-UPLC	Size-Exclusion Ultra-Performance Liquid Chromatography
TCA	Time-Corrected Area
USP	United States Pharmacopoeia
VEGF	Vascular Endothelial Growth Factor
