# Supplementary material for: Assessing the Comparability of Degradation Profiles Between Biosimilar and Originator Anti-VEGF Monoclonal Antibodies Under Thermal Stress
Source: Pharmaceuticals (Basel). 2025 Aug 26;18(9):1267. doi: 10.3390/ph18091267 (PMC12472202; doi:10.3390/ph18091267)
Supplement: Supplementary file 1 [file pharmaceuticals-18-01267-s001.zip › pharmaceuticals-3791479-supplementary.pdf]

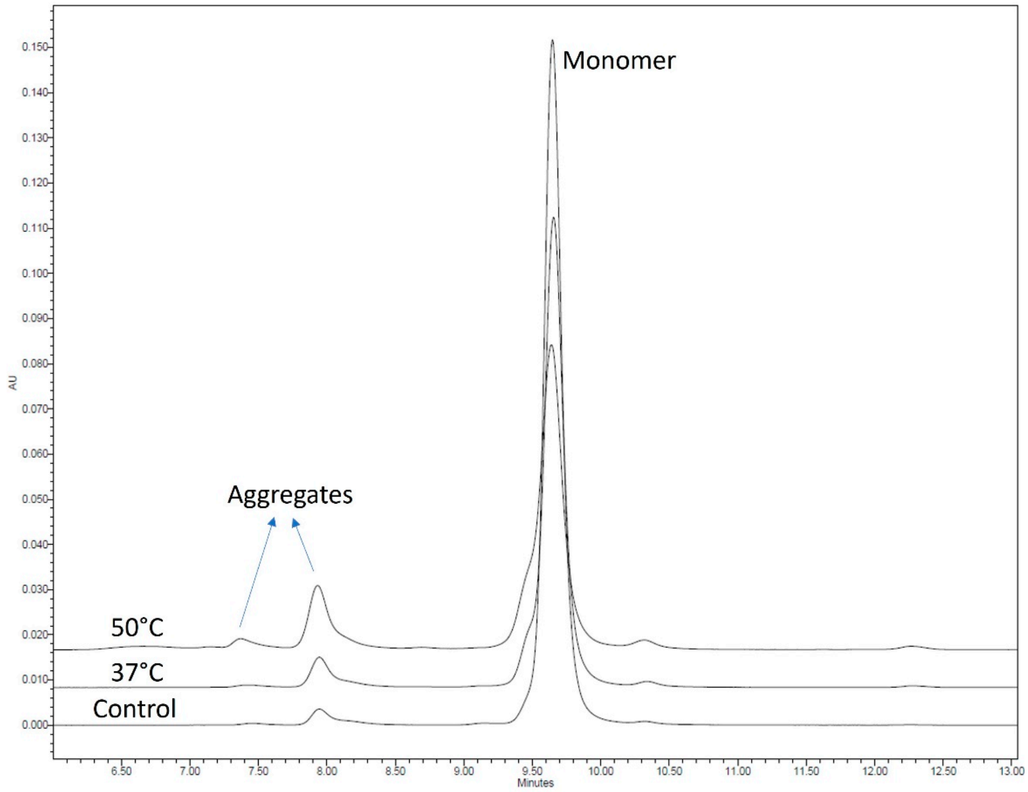

Figure S1. Overlay of SE-UPLC Chromatograms of OR-US Sample under Thermal Stress

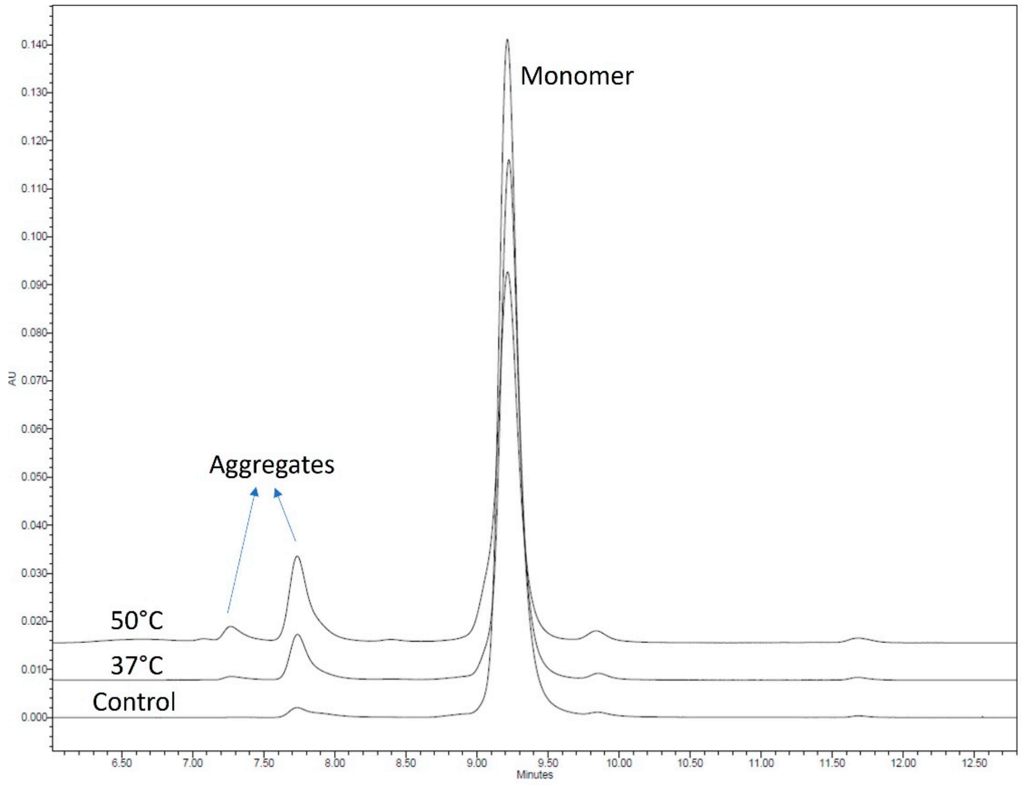

Figure S2. Overlay of SE-UPLC Chromatograms of BS Sample under Thermal Stress

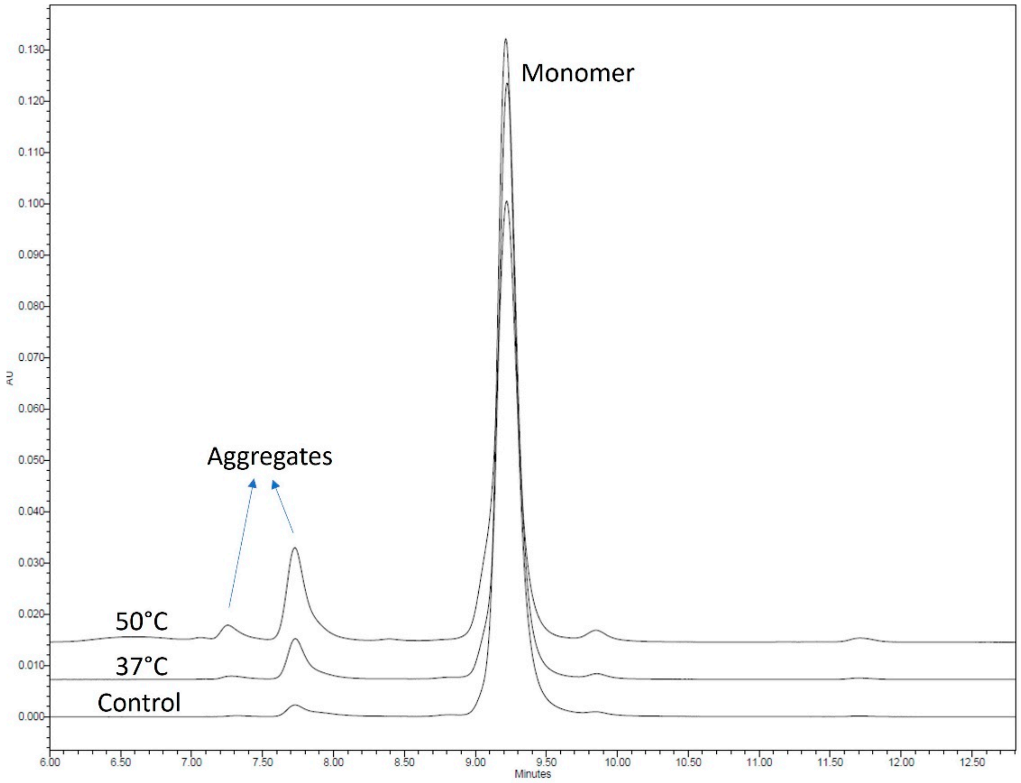

Figure S3. Overlay of SE-UPLC Chromatograms of OR-EU Sample under Thermal Stress

Table S1. Comparison of Expected and Observed Masses for Modified HC:T1 Peptide in Samples Incubated at 37°C. Modification site is shown in bold red font.

Sample Name	Peptide Sequence (Chain: Peptide No)	Expected Mass (Da)	Observed Mass (Da)
OR-US	**E**VQLVESGGGLVQPGGSLR (HC:T1)	1863.9923	1863.9888
BS			1863.9937
OR-EU			1863.9929

Table S2. Comparison of Expected and Observed Masses for Modified HC:T1 Peptide in Samples Incubated at 50°C. Modification site is shown in bold red font.

Sample Name	Peptide Sequence (Chain: Peptide No)	Expected Mass (Da)	Observed Mass (Da)
OR-US	**E**VQLVESGGGLVQPGGSLR (HC:T1)	1863.9923	1863.9916
BS			1863.9833
OR-EU			1863.9846

Table S3. Comparison of Expected and Observed Masses for Modified HC:T39 Peptide in Samples Incubated at 37°C. Modification sites are shown in bold red font.

Sample Name	Peptide Sequence (Chain: Peptide No)	Expected Mass (Da)	Observed Mass (Da)
OR-US	GFYPSDIAVEWES**N**GQPE**NN**YK (HC:T39)	2545.1154	2545.1175
BS			2545.1299
OR-EU			2545.1251

Table S4. Comparison of Expected and Observed Masses for Modified HC:T39 Peptide in Samples Incubated at 50°C. Modification sites are shown in bold red font.

Sample Name	Peptide Sequence (Chain: Peptide No)	Expected Mass (Da)	Observed Mass (Da)
OR-US	GFYPSDIAVEWES**N**GQPE**NN**YK (HC:T39)	2545.1154	2545.1171
BS			2545.1150
OR-EU			2545.1222
